# Extracts from the Leaves of *Cissus verticillata* Ameliorate High-Fat Diet-Induced Memory Deficits in Mice

**DOI:** 10.3390/plants10091814

**Published:** 2021-08-31

**Authors:** Woosuk Kim, Hyun Jung Kwon, Hyo Young Jung, Soon-Sung Lim, Beom-Goo Kang, Yong-Bok Jo, Dong-Sool Yu, Soo Young Choi, In Koo Hwang, Dae Won Kim

**Affiliations:** 1 Department of Biomedical Sciences, Research Institute for Bioscience and Biotechnology, Hallym University, Chuncheon 24252, Korea; wskim0503@konkuk.ac.kr (W.K.); donuts25@hallym.ac.kr (H.J.K.); sychoi@hallym.ac.kr (S.Y.C.); 2 Department of Anatomy, College of Veterinary Medicine, Veterinary Science Research Institute, Konkuk University, Seoul 05030, Korea; 3 Department of Biochemistry and Molecular Biology, Research Institute of Oral Sciences, College of Dentistry, Gangneung-Wonju National University, Gangneung 25457, Korea; 4 Department of Veterinary Medicine, Institute of Veterinary Science, Chungnam National University, Daejeon 34134, Korea; hyjung@cnu.ac.kr; 5 Department of Food Science and Nutrition, Institute of Korean Nutrition, Hallym University, Chuncheon 24252, Korea; limss@hallym.ac.kr; 6 Department of Biochemistry, College of Medicine, Hallym University, Chuncheon 24252, Korea; kbgda87@naver.com; 7 Department of Convergence Technology, Graduate School of Venture, Hoseo University, Seoul 06724, Korea; korjyb59@korea.ac.kr; 8 Department of Venture Management Graduate School of Venture, Hoseo University, Seoul 06724, Korea; sdt501@korea.ac.kr; 9 Department of Anatomy and Cell Biology, Research Institute for Veterinary Science, College of Veterinary Medicine, Seoul National University, Seoul 08826, Korea

**Keywords:** obesity, lipid profile, novel object recognition, neurogenesis, brain-derived neurotrophic factor

## Abstract

We investigated the effects of *Cissus verticillata* leaf extract (CVE) on a high-fat diet (HFD)-induced obesity and memory deficits. Male mice (5 weeks of age) were fed vehicle (distilled water), or 30, 100, or 300 mg/kg of CVE once a day for 8 weeks with an HFD. Treatment with CVE resulted in lower body weight and glucose levels in a concentration- and feeding time-dependent manner. LDL cholesterol and triglyceride levels were significantly lower in the CVE-treated HFD group than in the vehicle-treated HFD group. In contrast, high-density lipoprotein cholesterol levels did not show any significant changes. Lipid droplets and ballooning were reduced depending on the concentration of CVE treatment compared to the HFD group. Treatment with CVE ameliorated the increase in glucagon and immunoreactivities in the pancreas, and novel object recognition memory was improved by 300 mg/kg CVE treatment compared to the HFD group. More proliferating cells and differentiated neuroblasts were higher in mice treated with CVE than in vehicle-treated HFD-fed mice. Brain-derived neurotrophic factor (BDNF) levels were significantly decreased in the HFD group, which was facilitated by treatment with 300 mg/kg CVE in hippocampal homogenates. These results suggest that CVE ameliorates HFD-induced obesity and memory deficits in mice, associated with increased BDNF levels in the hippocampus.

## 1. Introduction

Obesity is induced by excessive fat accumulation due to a chronic imbalance between energy expenditure and energy intake. Obesity-induced hyperglycemia leads to cellular senescence in various tissues such as the liver, kidney, and pancreas by oxidative stress and chronic inflammation [1,2]. In addition, obesity-induced hyperglycemia can cause insulin resistance, a condition induced by the impairment of insulin-induced glucose uptake in insulin-sensitive tissues [3]. Insulin secretion is increased in the β-cells of pancreatic islets cells because of low insulin bioavailability in insulin-sensitive tissues [4]. In addition, several lines of evidence demonstrate the possible relationships between obesity and hippocampal function, including cognition in humans [5] and experimental animals [6,7]. Diabetic animals show a reduction in spatial memory with impaired insulin sensitivity [8] and decreased synaptic function in the hippocampus [9]. In humans, hyperglycemia affects memory performance and the hippocampal microstructure [10]. It has been reported that C57BL/6, AKR, and DBA/2 mice are highly susceptible to high-fat diet (HFD)-induced obesity. In contrast, C3H/He, BALB/cBy, and C57L mice are relatively resistant to HFD-induced obesity [11]. In a previous study we conducted with our colleagues, it was demonstrated that HFD-induced obesity in C57BL/6 mice significantly decreased proliferating cells and differentiated neuroblasts in the dentate gyrus compared to other strains (C3H/He) [12]. In addition, peroxisome proliferator-activated receptor γ (PPAR-γ) is one of the most important factors in regulating adipogenesis and is used to treat diabetes [13]. In addition, PPAR-γ agonists ameliorate HFD-induced neuronal impairment and neurogenesis in the hippocampus [14,15,16]. However, few systemic studies have attempted to search for therapeutics for lowering obesity-induced phenotypes in the blood, liver, pancreas, and brain.

Phytomedicine, or herbal medicine, can mitigate chronic diseases by using plants and herbs [17]. However, many herbs have risks despite being considered “natural” [18]. Nonetheless, herbal medicine is attractive for various metabolic diseases, including obesity, owing to its broad range of safety margins. *Cissus* spp., belonging to the family *Vitaceae*, consists of approximately 80 species [19] and has been used to treat diabetes, inflammation, epilepsy, and pain in traditional medicine [20,21,22,23,24]. Among *Cissus* spp., *Cissus verticillata* (also called *Cissus sicyoides*) and *Cissus quadrangularis* are the most popular; they decrease fat accumulation and weight gain [25,26,27,28]. In addition, *Cissus* spp. act centrally and lead to anticonvulsant, anxiolytic, and analgesic activity via the modulation of antioxidant capacity and GABA levels in the brain [29,30,31]. Although their importance lies in their role as a remedy for diabetes, most studies have been conducted on animals with type 1 diabetes using streptozotocin or alloxan [25,32]. In addition, there is no evidence of the effects of *Cissus verticillata* extract on obesity-induced memory impairment in mice.

This study examined the effects of extracts from *Cissus verticillata* (CVE) leaves on HFD-induced phenotypes, including body weight, blood glucose levels, serum parameters, liver morphology with hematoxylin, and eosin staining, and insulin and glucagon cells in the pancreas with immunohistochemical staining. In addition, we investigated novel object recognition memory and proliferating cells and differentiated neuroblasts using immunohistochemical staining for Ki67 and doublecortin (DCX), respectively. 

## 2. Results

### 2.1. Effect of CVE on Body Weight, Blood Glucose, and HbA1c Levels

**Figure 1:**
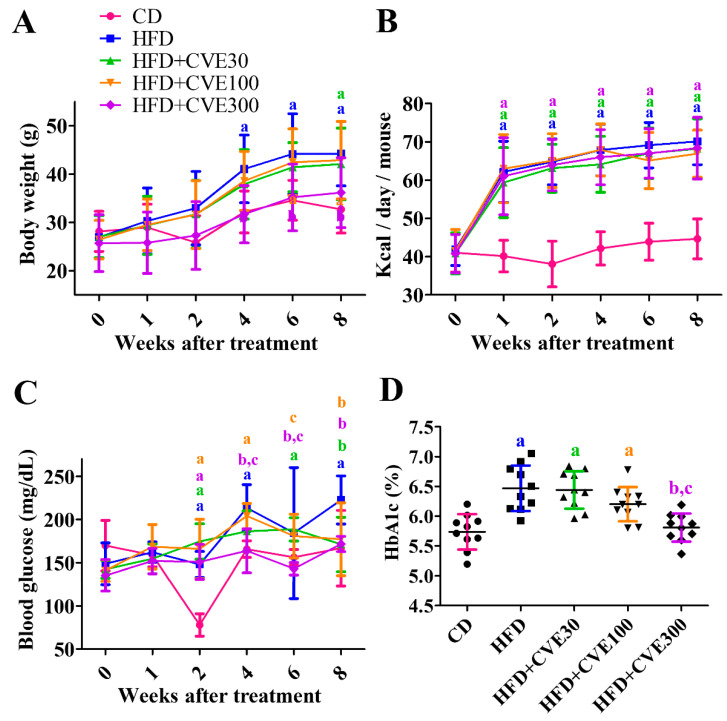
Changes in body weight (**A**), calorie intake (**B**), and blood glucose levels (**C**) during experiments as well as HbA1c levels 8 weeks after treatment (**D**) in the control diet (**CD**) and high fat diet-fed mice with the vehicle, or 30, 100, and 300 mg/kg *C. verticillate* L. extracts (HFD, HFD+CVE30, HFD+CVE100, and HFD+CVE300, respectively). The data were analyzed by one-way analysis of variance for HbA1c and two-way analysis of variance for others followed by Bonferroni’s post hoc test (^a^ *p* < 0.05, significantly different from the CD group; ^b^ *p* < 0.05, significantly different from the HFD group; ^c^ *p* < 0.05, significantly different from the HFD+CVE30 group). The bars indicate the mean ± standard deviation. CD, control diet; CVE, *Cissus verticillata* leaf extract; HFD, high-fat diet.

In all groups, body weight increased with time after HFD feeding and/or CVE treatment. In particular, body weight was significantly higher in the HFD group than in the control diet (CD) group at 4 weeks after the experiment. In the HFD+CVE30 group, the body weight was significantly higher at 8 weeks after the experiment than in the CD group. In the HFD+CVE100 group, the body weight was significantly higher at 6 weeks after the experiment than in the CD group. In the 300 mg/kg CVE-treated (HFD+CVE300) group, there were no significant differences in body weight compared to the CD group depending on time after the experiment, and the body weight in the HFD+CVE300 group was significantly lower at 4 weeks after the experiment compared to the HFD group. After that, the body weight was maintained at significantly lower levels at 8 weeks after the experiment. Two-way analysis of variance (ANOVA) showed no significant interactions (F = 1.18, DFn = 20, DFd = 270, *p* = 0.2751) with body weight between time after treatment and diet/extract treatment (Figure 1A).

Calorie intake was significantly higher in the HFD group than in the CD group from 1 week after HFD feeding. In the HFD+CVE30, HFD+CVE100, and HFD+CVE300 groups, calorie intake was not significantly different from HFD group. Two-way ANOVA showed that calorie intake significantly interacted (F = 4.51, DFn = 20, DFd = 270, *p* < 0.0001) with time after treatment and diet/extract treatment (Figure 1B).

Blood glucose levels fluctuated, presumably because the mice did not fast. However, blood glucose levels in the HFD group tended to increase with time after HFD feeding, and the levels were significantly higher than those in the CD group from 2 weeks after treatment. In the HFD+CVE30 and HFD+CVE100 groups, blood glucose levels were significantly lower at 8 weeks after treatment compared to the HFD group. In contrast, in the HFD+CVE300 group, blood glucose levels were significantly lower at 4 weeks after treatment compared to the HFD group. Two-way ANOVA showed that the time after treatment significantly interacted (F = 6.04, DFn = 20, DFd = 270, *p* < 0.0001) in blood glucose levels with diet and extract treatment (Figure 1C).

Glycated hemoglobin (HbA1c) levels were measured 8 weeks after HFD feeding and/or CVE treatment. The HbA1c level was 5.737% in the CD group, significantly increasing to 6.467% in the HFD group. In the HFD+CVE30 and HFD+CVE100 groups, HbA1c levels were significantly higher than in the CD group, and there were no significant differences in HbA1c levels between HFD and HFD+CVE30 or HFD+CVE100 groups. In the HFD+CVE300 group, the HbA1c level was significantly lower than in the HFD and HFD+CVE30 groups, and there was no significant difference in HbA1c levels between the CD and HFD+CVE300 groups (Figure 1D).

### 2.2. Effect of CVE on Biochemical Parameters in the Serum

**Figure 2:**
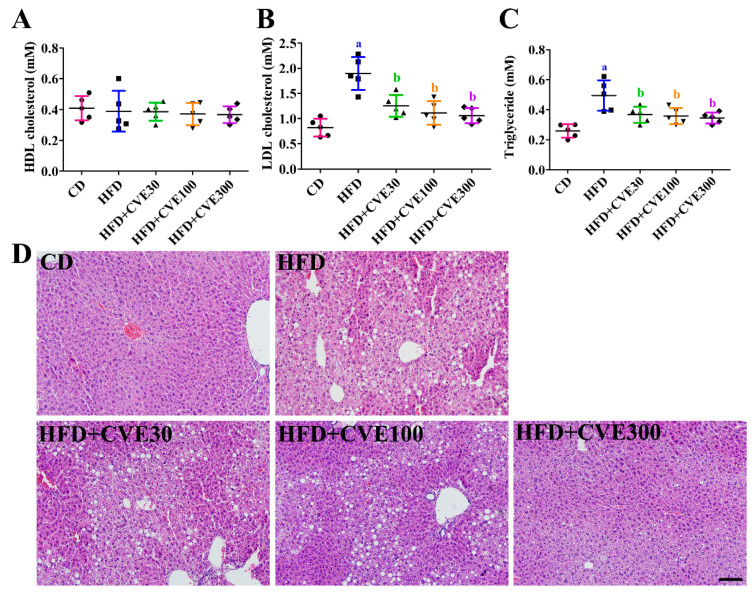
Lipid profiles such as high-density lipoprotein (HDL) cholesterol (**A**), low-density lipoprotein (LDL) cholesterol (**B**), and triglyceride (**C**), as well as the morphology of liver with hematoxylin & eosin staining (**D**) at 8 weeks after treatment in the CD, HFD, HFD+CVE30, HFD+CVE100, and HFD+CVE300 groups. The data were analyzed by one-way analysis of variance followed by Bonferroni’s post hoc test (^a^ *p* < 0.05, significantly different from the CD group; ^b^ *p* < 0.05, significantly different from the HFD group). The bars indicate the mean ± standard deviation. CD, control diet; CVE, *Cissus verticillata* leaf extract; HFD, high-fat diet.

High-density lipoprotein (HDL) cholesterol levels did not show any significant changes between groups (Figure 2A). Still, low-density lipoprotein (LDL) cholesterol levels were significantly higher in the HFD group than in the CD group (231.3 %). In the HFD+CVE30, HFD+CVE100, and HFD+CVE300 groups, LDL cholesterol levels were significantly decreased compared to the HFD group to 152.5%, 135.8%, and 129.3% of that in the CD group, respectively (Figure 2B).

In addition, triglyceride levels were significantly increased in the HFD group to 191.6% of that in the CD group. In the HFD+CVE30, HFD+CVE100, and HFD+CVE300 groups, triglyceride levels were significantly lower than those in the HFD group (141.9%, 138.3%, and 133.4% of that in the CD group, respectively) (Figure 2C).

### 2.3. Effect of CVE on Liver Morphology

In the CD group, hematoxylin and eosin staining showed few ballooning and lipid droplets in the liver’s hepatocytes. However, in the HFD group, staining revealed ballooning of hepatocytes and accumulation of lipid droplets. In addition, strong eosinophilic cells were observed in the liver. In the HFD+CVE30, HFD+CVE100, and HFD+CVE300 groups, ballooning of hepatocytes was observed, and there was almost no ballooning of hepatocytes and accumulation of lipid droplets in the HFD+CVE300 group (Figure 2D).

### 2.4. Effect of CVE on Insulin and Glucagon Secreting Cells in the Pancreas

**Figure 3:**
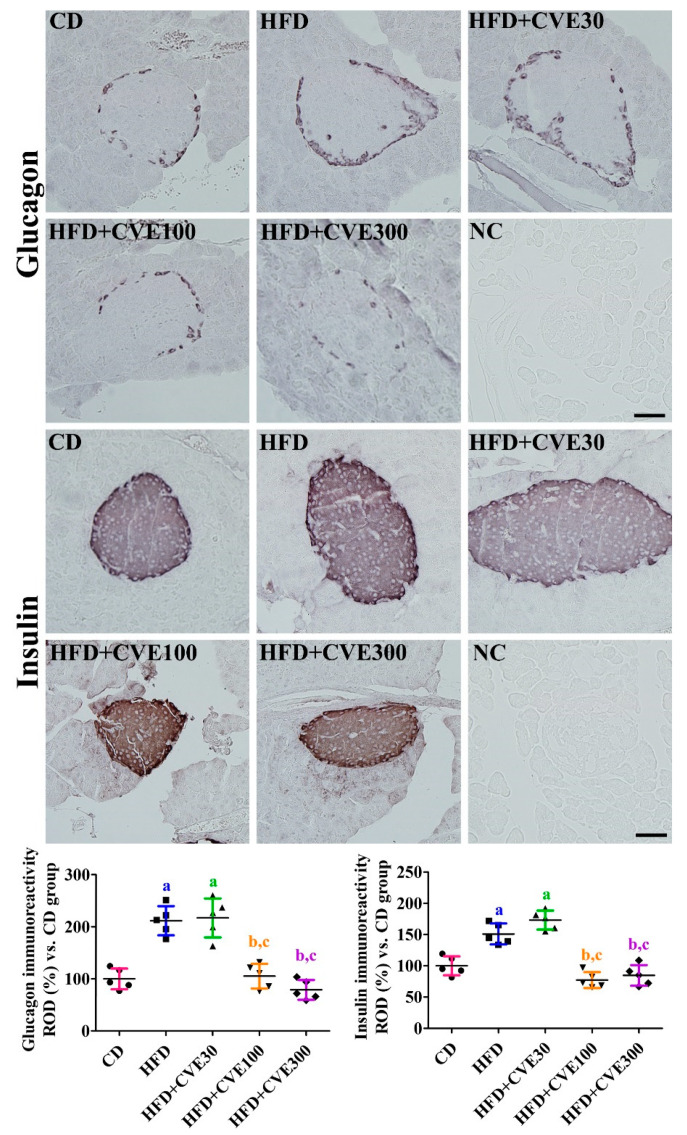
Immunohistochemical staining for glucagon and insulin in the pancreas of mice in CD, HFD, HFD+CVE30, HFD+CVE100, and HFD+CVE300 groups at 8 weeks after treatment. Optical density was calculated based on pixel number with gray density, and ROD is demonstrated vs. the CD group. The data were analyzed by one-way analysis of variance followed by Bonferroni’s post hoc test (^a^ *p* < 0.05, significantly different from the CD group; ^b^ *p* < 0.05, significantly different from the HFD group; ^c^ *p* < 0.05, significantly different from the HFD+CVE30 group). The bars indicate the mean ± standard deviation. CD, control diet; CVE, *Cissus verticillata* leaf extract; HFD, high-fat diet; NC, negative control staining; ROD, relative optical density.

In all groups, insulin-immunoreactive cells were abundantly detected in the islets of the pancreas, while glucagon-immunoreactive cells were found in the periphery of the islet. There were no significant differences in the distribution patterns of insulin- and glucagon-immunoreactive cells. However, glucagon immunoreactive cells were abundant in the HFD group, and glucagon immunoreactivity was significantly increased to 211.7% in the CD group. In the HFD+CVE30, HFD+CVE100, and HFD+CVE300 groups, glucagon immunoreactivity decreased in a concentration-dependent manner in the islets to 217.1%, 105.4%, and 79.0%, respectively, compared to the CD group. In addition, insulin-immunoreactive islets were larger in the HFD and HFD+CVE30 groups than in the CD group, and insulin immunoreactivity was 151.0% and 173.1%, respectively, for those two groups. In the HFD+CVE100 and HFD+CVE300 groups, insulin immunoreactivity was significantly decreased to 77.3% and 84.8%, respectively, of that in the CD group, compared to the HFD group (Figure 3).

### 2.5. Effect of CVE on Novel Object Recognition Memory

**Figure 4:**
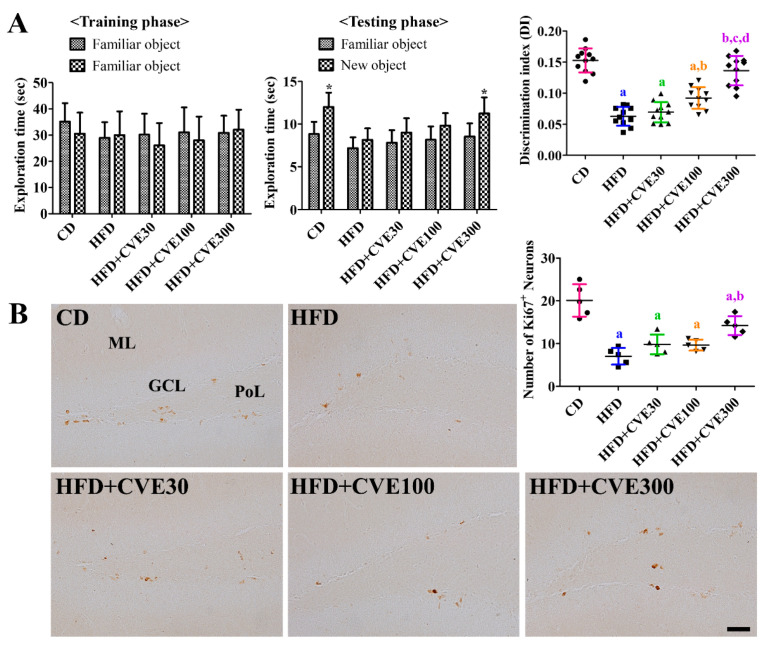
Exploration time for familiar and familiar or new (novel) objects during training and testing phase as well as calculated discrimination index in the testing phase (**A**) as well as immunohistochemical staining for Ki67 (**B**) in the CD, HFD, HFD+CVE30, HFD+CVE100, and HFD+CVE300 groups at 8 weeks after treatment. The number of Ki67-immunoreactive neurons was counted in the dentate gyrus per section. The data were analyzed by two-way analysis of variance for exploration time and one-way analysis of variance followed by Bonferroni’s post hoc test for others (* *p* < 0.05, significantly different from the familiar object; ^a^ *p* < 0.05, significantly different from the CD group; ^b^ *p* < 0.05, significantly different from the HFD group; ^c^ *p* < 0.05, significantly different from the HFD+CVE30 group; ^d^ *p* < 0.05, significantly different from the HFD+CVE100 group). The bars indicate the mean ± standard deviation. CD, control diet; CVE, *Cissus verticillata* leaf extract; HFD, high-fat diet.

In the training phase, there were no significant differences in the exploration time among the groups. In addition, a two-way ANOVA test showed that there were no significant interactions (F = 0.62, DFn = 4, DFd = 90, *p* = 0.6512) between the object and exploration time. However, mice in the CD group explored novel objects significantly more than familiar ones in the testing phase. The discrimination index was 0.153, although the interaction between the object and exploration time was not statistically significant (F = 1.98, DFn = 4, DFd = 90, *p* = 0.1038). In the HFD group, mice explored the familiar and novel objects for a similar time, and the discrimination index was significantly decreased to 41.2% of that in the CD group. In the HFD+CVE30, HFD+CVE100, and HFD+CVE300 groups, the time spent exploring the novel object was increased in a concentration-dependent manner, and the mice in the HFD+CVE300 group spent significantly more time exploring the novel object than the HFD group. Similarly, the discrimination index increased concentration-dependent and was highest in the HFD+CVE300 group, 89.3% of that in the CD group (Figure 4A).

### 2.6. Effect of CVE on Proliferating Cells and Differentiated Neuroblasts in the Dentate Gyrus

In the CD group, Ki67-immunoreactive nuclei were mainly observed in the subgranular zone of the dentate gyrus (20.1/section). In the HFD group, fewer Ki67-immunoreactive nuclei were detected in the dentate gyrus, and the number was significantly decreased to 35.1% of that in the CD group. In the HFD+CVE30, HFD+CVE100, and HFD+CVE300 groups, more Ki67-immunoreactive nuclei were found in the dentate gyrus in proportion to the concentration of CVE. In particular, the number was significantly higher in the HFD+CVE group than in the HFD group and was 70.7% of that in the CD group (Figure 4B).

**Figure 5:**
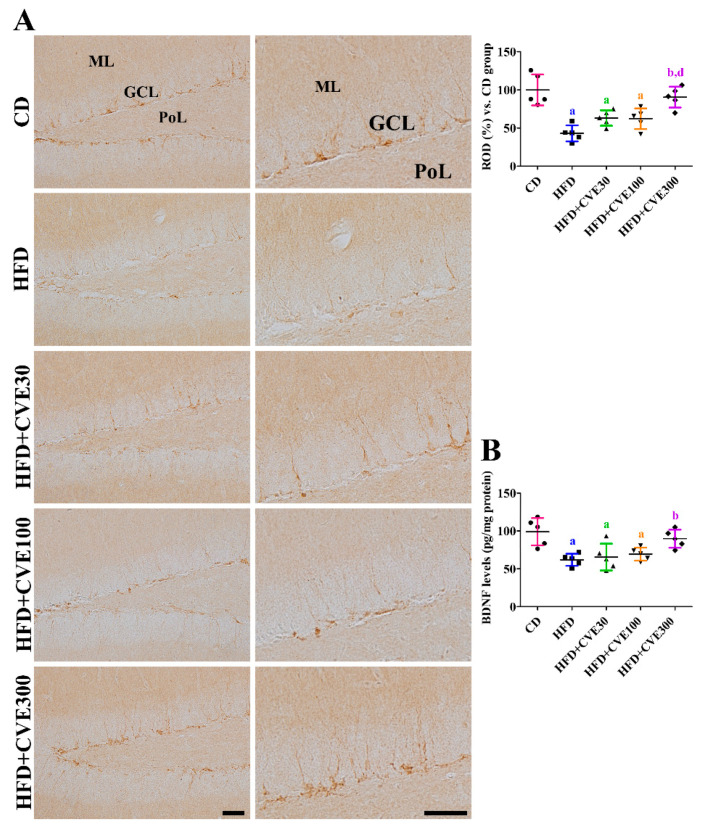
Immunohistochemical staining for DCX (**A**) and measurement of BDNF (**B**) in the CD, HFD, HFD+CVE30, HFD+CVE100, and HFD+CVE300 groups at 8 weeks after treatment. Optical density was calculated based on pixel number with gray density, and ROD is demonstrated vs. the CD group. The data were analyzed using one-way analysis of variance followed by Bonferroni’s post hoc test (^a^ *p* < 0.05, significantly different from the CD group; ^b^ *p* < 0.05, significantly different from the HFD group; ^d^ *p* < 0.05, significantly different from the HFD+CVE100 group). The bars indicate the mean ± standard deviation. CD, control diet; CVE, *Cissus verticillata* leaf extract; DCX, doublecortin; HFD, high-fat diet; ROD, relative optical density.

DCX immunoreactivity was observed in the cytoplasm and dendrites of the dentate gyrus. In the HFD group, fewer DCX immunoreactive structures were observed in the dentate gyrus, and DCX immunoreactivity was significantly decreased to 43.1% of that in the CD group. In the HFD+CVE30, HFD+CVE100, and HFD+CVE300 groups, more DCX immunoreactive cells and dendrites were detected in the dentate gyrus; however, DCX immunoreactivity was significantly increased only in the HFD+CVE300 group (Figure 5A).

### 2.7. Effect of CVE on Brain-Derived Neurotrophic Factor (BDNF) Levels in the Hippocampus

In the HFD group, BDNF levels in hippocampal homogenates were significantly decreased to 62.5% of those in the CD group. In the HFD+CVE30 and HFD+CVE100 groups, BDNF levels were similar to those in the HFD group. In the HFD+CVE300 group, BDNF levels were significantly increased compared to those in the HFD group and were 90.7% of those in the CD group (Figure 5B).

## 3. Discussion

HFD-induced obesity causes metabolic complications, including insulin resistance [33,34], facilitating cellular senescence in various tissues with inflammation and oxidative stress [1,2]. The present study observed that body weight, calorie intake, and blood glucose levels significantly increased with HFD feeding time after treatment in C57BL/6 mice, a strain susceptible to HFD-induced obesity. In particular, blood glucose levels fluctuated and decreased in the CD group at 2 weeks after the experiments. We observed lower glucose levels than that at 0 weeks after the experiment in this period, although statistical significance was not detected. This may be due to a decrease in calorie intake and body weight. In the present study, the lipid profile showed significant increases in LDL cholesterol and triglyceride levels. CVE treatment ameliorated obesity-induced increases in body weight, blood glucose levels, and lipid profiles, although CVE did not affect calorie intake. This result is consistent with a previous study showing that leafy stem extracts from *Cissus polyantha* decreased dexamethasone-induced hyperglycemia and lipidemia in rats [24]. *Cissus adnate* Roxb. has the potential to lower blood glucose levels [35]. In addition, CVE decreased blood glucose and lipid profiles in streptozotocin- and alloxan-induced diabetes [26]. In this study, we confirmed HbA1c levels in the blood because it is an important indicator for the evaluation of diabetes [36,37]. HFD-induced obesity significantly increased HbA1c levels, and this result was consistent with previous studies that showed that HFD feeding significantly increased HbA1c levels [38,39]. Treatment with CVE ameliorated HFD-induced HbA1c levels, suggesting the antidiabetic effects of CVE in mice. However, conflicting evidence has been reported that decoction of *C. verticillata* L. lowered blood glucose levels but had no or minimal effects on lipid profiles in streptozotocin-induced diabetic rats [25]. This discrepancy may be associated with the concentration of the CVE used in the study. In the present study, CVE exerted a strong effect at 300 mg/kg of CVE. However, in the present study, we only observed the HbA1c in the blood and HDL cholesterol, LDL cholesterol, and triglyceride levels at sacrifice time, not at various time points after treatment, such as with body weight and blood glucose levels, because we could obtain a low volume of blood at various time points in mice. The baseline and change-to-change comparisons needed to be measured to confirm the possible mechanisms of action of CVE in HFD-fed mice.

HFD-fed mice showed ballooning and accumulation of lipid droplets in hepatocytes [40] and increased β-cell mass to compensate for the high demand of insulin. In the present study, we confirmed morphological changes in hepatocytes to support the biochemical results. We observed that treatment with CVE reduced HFD-induced hepatocyte ballooning and accumulation of lipid droplets. In addition, CVE treatment significantly mitigated the HFD-induced increase in insulin and glucagon immunoreactivity in pancreatic islets. *Cissus quadrangularis* stem extract and its ethyl decreased hepatocellular damage and lipid deposition in high-fat and high-fructose diet-fed rats [41] and inflammatory responses in nicotinamide/streptozotocin-induced type 2 diabetic rats [42]. However, unlike our study, Lekshmi et al. observed that the nicotinamide/streptozotocin model decreased islet size, and the ethyl acetate fraction of *Cissus quadrangularis* stem extract decreased pancreatic β-cell damage [42]. In the present study, we observed significant increases in insulin and glucagon immunoreactivity in pancreatic islets, which may be associated with increased β-cell mass and pancreatic islet size.

We also observed hippocampal function based on a novel object recognition test and neurogenesis because HFD-induced obesity exhibited impairment of cognitive function [6,7] and decreased neurogenesis in the dentate gyrus [16,43]. In addition, few studies have reported the effects of *Cissus* spp. on neurological diseases and brain function. Treatment with CVE significantly improved HFD-induced impairment in novel object recognition memory and decreased Ki67-immunoreactive proliferating cells and DCX-immunoreactive differentiated neuroblasts. Moto et al. demonstrated the central effects of *C. quadrangularis* extract against status epilepticus [31]. To elucidate the possible mechanisms of CVE against HFD-induced memory impairments and decreases in neurogenesis, BDNF levels were measured in hippocampal homogenates because HFD-induced obesity decreased BDNF levels, which play important roles in memory formation [44] and adult neurogenesis [45]. BDNF levels were suppressed in the brain during HFD feeding [46] and correlated with impaired cognitive performance [47]. In BDNF heterozygous mice, HFD feeding resulted in a more prominent loss of synaptic proteins such as synaptosome-associated protein 25 and postsynaptic density 95 in the cerebral cortex than wild-type mice [48]. HFD-fed mice showed significantly lower levels of BDNF in hippocampal homogenates than the CD group, and 300 mg/kg CVE treatment significantly ameliorated the reduction in BDNF levels. This result suggests that CVE ameliorates the HFD-induced reduction in proliferating cells and differentiated neuroblasts. This may be associated with increased BDNF levels in the hippocampus. *C. quadrangularis* contains quercetin and ascorbic acid [49], which have therapeutic effects against HFD-induced liver damage [43], heart damage [50], and damage to blood vessels [51]. However, the analysis of functional compounds from CVEs remains to be elucidated.

In conclusion, HFD-fed mice showed increased body weight, blood glucose levels, lipid profiles in serum, and ballooning and accumulation of lipids in hepatocytes. In addition, HFD-fed mice showed a decrease in the number of proliferating cells and differentiated neuroblasts in the dentate gyrus by downregulation of BDNF. Treatment with CVE ameliorated HFD-induced metabolic changes, impaired hippocampal function, and neurogenesis. This beneficial effect may be associated with increases in BDNF levels in the hippocampus, and CVE could be a candidate to reduce HFD-induced problems in the liver, pancreas, and hippocampus.

## 4. Materials and Methods

### 4.1. Preparation of CVE

Fresh leaves of *Cissus verticillata* L. were purchased from the local market in Ecuador, collected in November 2016. The taxonomic determination was made by Emeritus Prof. H.J. Chi, Seoul National University, South Korea. The voucher species, HL201805, was deposited at the RIC herbarium at Hallym University.

The CVE was obtained as previously described [52]. Briefly, the leaves were dried at 60 °C for 48 h and the dried leaves were homogenized for 2 min to the smallest possible particle size (approximately 200 μm) using a blender, and extracted (500 g/5 L) using the water extraction method (100 °C for 3 h) with a reflux condenser. The extract was then freeze-dried at −50 °C for 24 h. The extract (75 g, 15% yield) was dissolved in ethanol (1 L) and centrifuged to the ethanol-soluble fraction, which (15 g, 20% yield from water extract) was evaporated to dryness by rotary evaporation.

### 4.2. Experimental Animals

Fifty C57BL/6 mice (5 weeks of age) were purchased from Orient Bio (Seongnam, Korea) and housed in a specific pathogen-free facility at the College of Veterinary Medicine of Seoul National University. All mice were housed in polycarbonate cages (3–4 animals per cage) in individually ventilated caging systems with a plastic mouse igloo. The temperature (22 ± 2 °C) and relative humidity (50–60%) in the animal room were tightly controlled by an automatic regulated system. The experimental procedures were approved by the Institutional Animal Care and Use Committee of Seoul National University (SNU-170630-8-2).

### 4.3. Experimental Groups

The animals were categorized into two groups: CD (*n* = 10) and HFD-fed (*n* = 40) mice. The latter group was further divided into four subgroups as follows: HFD (*n* = 10), HFD + CVE30 (*n* = 10), HFD + CVE100 (*n* = 10), and HFD + CVE300 (*n* = 10) groups. CVE was dissolved in distilled water. After one week of acclimatization, a control chow or high-fat diet (D12492i, Research Diets) was fed to the mice. CVE (180 mg) was dissolved in 6 mL distilled water for a stock solution and further diluted with distilled water to adjust the final concentration of CVE in each mouse. Finally, 30, 100, and 300 mg/kg CVE (0.3 mL) were orally administered to mice using a feeding needle once daily for 8 weeks.

### 4.4. Novel Object Recognition Memory

Following HFD feeding with or without CVE treatment, hippocampus-independent memory was assessed using a novel object recognition test. On day 55 of the treatment, animals were placed in a novel object recognition chamber (25 × 25 × 25 cm^3^) for adaptation (2 min). On day 56 of the treatment, they were freed to explore the same two objects located at opposite corners for the training phase for 5 min. Twenty-four hours after the training phase, one object was replaced with a novel object at the same location for 5 min. Exploration activity was defined as the nose of mice located within 2 cm of the object, and the total exploration time was measured and calculated as described in a previous study [53]. The discrimination index was calculated as the ratio of time differences to the total exploration time between the novel and familiar objects. In every trial, familiar and novel objects were cleaned with bleach to remove residual odors.

### 4.5. Biochemical Experiments and Morphological Study of Peripheral Tissues

Following HFD feeding with or without treatment with CVE, animals (*n*  =  5 in each group) and diets were weighed, and blood was obtained from the caudal vein to measure the glucose levels at 1, 2, 4, 6, and 8 weeks after treatment. Caloric uptake was calculated by multiplying the consumed food in grams by the calories per gram in the diets. Animals were sacrificed with a mixture of alfaxalone (Alfaxan, 75 mg/kg; Careside, Seongnam, South Korea) and xylazine (10 mg/kg; Bayer Korea, Seoul, South Korea), and blood, liver, brain, and pancreas were collected. Blood glucose and HbA1c levels were measured using a blood glucose monitor (Ascensia Elite XL Blood Glucose Meter, Bayer, Toronto, ON, Canada) and an HbA1c analyzer (Tosoh, Ayase, Japan), as demonstrated in a previous study [54]. Plasma was separated as described previously [54]. Serum parameters, such as LDL cholesterol (cat. no. ab65390, Abcam, Cambridge, UK), HDL cholesterol (cat. no. ab65390, Abcam), and triglycerides (cat. no. ab65336, Abcam) levels were measured using enzyme-linked immunosorbent assay (ELISA) kits according to the instructor’s guidelines. In addition, both hippocampi were dissected from the brain, and BDNF levels were measured in both hippocampi using an ELISA kit (cat. no. DY248, R&D Systems, Minneapolis, MN, USA) as described previously [16].

Tissues, such as the liver and pancreas, were processed for paraffin-embedded sections. Paraffin-embedded tissues were cut into 3 μm sections and mounted on silane-coated slides. Hematoxylin and eosin staining was conducted in the liver tissue, and immunohistochemical staining for insulin and glucagon was performed in the pancreas as described previously. Sections were rehydrated, and the sections were exposed to heat with citrate buffer (pH 6.0) in a 2100-retriever (Prestige Medical, Lancashire, UK) to retrieve the antigenicity of insulin and glucagon. Thereafter, sections were treated with 0.3% hydrogen peroxide solution to remove endogenous peroxidase and were incubated with 5% normal goat serum to block nonspecific binding at 25 °C for 1 h. Sections were incubated with rabbit anti-insulin (1:100, cat. no. LS-B2526, LSBio, Seattle, WA, USA) or antiglucagon (1:200, cat. no. LS-B8235, LSBio) overnight at 25 °C. Thereafter, the sections were treated with goat antirabbit IgG (1:200, cat. no. BA-1000-1.5, Vector, Burlingame, CA, USA) and peroxidase-conjugated streptavidin (1:200, cat. no. SA-5004-1, Vector) for 2 h at 25 °C. Finally, the sections were visualized with 3,3-diaminobenzidine tetrachloride (Sigma-Aldrich, St. Louis, MO, USA) intensified with nickel. All antibodies were diluted using 0.1 M PBS containing 5% goat serum (Vector) and 0.02% Triton X-100 (Sigma). The specificity of antibodies such as insulin and glucagon was confirmed by substituting goat antirabbit IgG with isotype control IgG (Abcam) [55].

### 4.6. Morphological Study in the Hippocampus

Following HFD feeding with or without treatment with CVE, animals (*n* = 5 in each group) were anesthetized with a mixture of alfaxalone (Careside) and xylazine (Bayer Korea) and perfused transcardially with physiological saline and 4% paraformaldehyde. Brain samples were obtained and cryofrozen to cut into 30 μm sections located between 1.82 and 2.30 mm caudal to the bregma [56]. Four sections, each 120 μm apart, were selected, and immunohistochemical staining was conducted using rabbit antiKi67 antibody (1:1000; cat. no. ab16667; Abcam) or rabbit antiDCX antibody (1:5000; cat. no. ab18723, Abcam) to detect proliferating cells and differentiated neuroblasts, respectively, in the dentate gyrus, as described above and previously [53,57]. The specificity of Ki67 and DCX was confirmed in a previous study [58].

### 4.7. Data Analysis

Insulin and glucagon immunoreactivity in the pancreas and DCX immunoreactivity in the dentate gyrus were semiquantified based on the sum of density in each pixel using ImageJ software version 1.80 (National Institutes of Health, Bethesda, MD, USA), as described previously. In addition, the number of Ki67-positive cells in the dentate gyrus was counted using OPTIMAS software (version 6.5; CyberMetrics^®^ Corporation, Phoenix, AZ, USA), as described in a previous study [53,57].

### 4.8. Statistical Analysis

Statistical analysis was performed using GraphPad Prism 5.01 software (GraphPad Software, Inc., La Jolla, CA, USA). Data were analyzed by two-way (for body weight, calorie intake, blood glucose level, and novel object recognition test) or one-way ANOVA followed by Bonferroni’s post hoc test with significance considered at *p* < 0.05.

## Data Availability

The datasets and supporting materials generated during and/or analyzed during the current study are available from the corresponding author upon reasonable request.
